# Evaluation of a web-based information platform on youth depression and mental health in parents of adolescents with a history of depression

**DOI:** 10.1186/s13034-023-00703-x

**Published:** 2024-01-13

**Authors:** Lucia Iglhaut, Regine Primbs, Sara Kaubisch, Chiara Koppenhöfer, Charlotte E. Piechaczek, Pia-Marie Keim, Maria Kloek, Lisa Feldmann, Gerd Schulte-Körne, Ellen Greimel

**Affiliations:** Department of Child and Adolescent Psychiatry, Psychosomatics and Psychotherapy, Hospital of the Ludwig-Maximilians-University (LMU) Munich, Nussbaumstraße 5, 80336 Munich, Germany

**Keywords:** Youth depression, Major depression, Knowledge, Web-based, Parents, Depression literacy, Ehealth

## Abstract

Research shows the important role of parents’ mental health literacy in detecting depressive symptoms and supporting their children to seek professional help. Improving mental health literacy in parents has recently gained even greater importance due to the negative impact of the COVID-19 pandemic on children and adolescents’ mental health. The aim of the present experimental pre-post-follow-up study was to examine knowledge change after the reception of contents from an innovative web-based platform (www.ich-bin-alles.de/eltern) containing evidence-based information on youth depression and mental health in parents of adolescents with a history of depression. A second aim was to assess evaluation of the layout and the acceptance of the platform. *N* = 33 parents of adolescents with a history of depression (either current or remitted depression) were presented different content domains of the website. Participants’ knowledge about depression was assessed at pre- and post-intervention, and at a four week follow-up. Moreover, parents evaluated the acceptance and the layout of the website. The trial was preregistered at clinicaltrials.gov (NCT05335564). The results showed a significant increase in total knowledge from pre to post, which remained stable over the course of four weeks. Explorative analyses showed that sociodemographic variables did not influence the extent of knowledge gain. Acceptance rates were high and evaluations of the website’s layout were positive. The findings show that the web-based information portal is a promising and appealing means to increase parental knowledge on youth depression. Low-threshold psychoeducational approaches like websites are particularly relevant in times of crisis and increased prevalence rates of depressive symptoms and disorders (ehealth). These results are an important basis for future studies as well as approaches that aim to impart knowledge about mental disorders like youth depression via web-based means. Furthermore, they bear implications for policy decisions concerning mental health education and campaigns.

**Supplementary Information:**

The online version contains supplementary material available at 10.1186/s13034-023-00703-x.

## Background

Depression is one of the most common mental disorders in childhood and adolescence [1], with incidences rising up to 7.5% [2]. In the context of the COVID-19 pandemic, recent studies report increased mental health problems in youth, including a heightened prevalence of depressive symptoms and depressive disorder. For example, a recent meta-analysis found an increase of clinically relevant depressive symptoms to 25.2% among children and adolescents [3]. Moreover, a systematic review reported an estimated increase of 27.6% for major depressive disorder among the global population in the context of the COVID-19 pandemic, with a more pronounced increase in younger compared to older age groups [4]. There is further evidence which shows that increases in depression symptoms and clinically relevant depression rates during the COVID-19 pandemic were related to pandemic-related restrictions, with more stringent and invasive pandemic-related restrictions being associated with higher effect estimates [5]. The same pattern was also evident for anxiety symptoms and clinically relevant anxiety rates [6].

Despite the high burden and negative psychosocial and medical consequences of youth depression [7, 8], data from Germany show that only 12.5% of adolescents with clinically relevant depressive symptoms seek help and start treatment in a 12-month period [9]. Children and adolescents and their families are confronted with a number of barriers when it comes to seeking help in case of mental problems including long waiting times and limited treatment offers, negative prior experiences, fear of stigmatisation and limited knowledge regarding treatment options [10–12]. In the context of depression, limited depression literacy is also seen as a barrier against seeking professional help [13]. Yet, reducing the time between the onset of a depressive disorder and the start of treatment is of great importance, as the longer a depressive disorder is not treated effectively, the higher the risk for chronification with more episodes throughout the course [14, 15].

Parents of youth with a mental disorder play an important role in supporting their children to seek professional help [16]. There is evidence that knowledge about mental disorders, symptom severity and treatment in parents of children with a history of depression is associated with more treatment-seeking, improved treatment decision and better treatment quality concerning their children [17, 18]. Further research shows that parents’ knowledge about, e.g., their child’s mental disorder influences the parental ability to carry out practices to support their affected offspring [19]. Thus, parental knowledge about mental disorders like youth depression is crucial for effective parental support. There is further evidence that parents of adolescents with mental disorders proactively seek sources to increase their knowledge in order to increase their support abilities [20], which underlines their willingness to provide support.

Thus, well-informed parents can substantially contribute to the early detection of mental disorders like depression in their children, to supporting them in seeking help from suitable specialists and to dealing with the challenges over the course of their illness. Yet, a majority of children and adolescents as well as their parents know only little about the symptoms, causes and forms of treatment of depression in childhood and adolescence [21, 22]. In a recent study [23], parents were asked to respond to vignettes about children and children with different mental health problems. The results showed less knowledge and more stigma in parents towards depression compared to other frequent mental health conditions like attention-deficit/hyperactivity disorder (ADHD). Another study investigated knowledge on mood disorders and their treatment in parents of children with a diagnosed mood disorder [24]. The results showed that parents had some basic knowledge about these topics, yet none had full knowledge, suggesting room for improvement. Together, these findings indicate the need to provide more psychoeducation offers to parents on depression in childhood and adolescence in order to enhance their depression literacy and reduce stigma towards the disorder.

Enhancing parents’ knowledge about depression in childhood and adolescence can be achieved by making such information broadly available, for instance with low-threshold online approaches. Up to now, there are only few broadly accessible evidence-based sources of information about youth depression for children and adolescents as well as their parents. Due to the increasing web usage [25–27], even more during the COVID-19 pandemic [28], digital sources of information are increasingly gaining importance. Digital formats on mental health topics are well accepted by adults of various ages [29, 30], yet scientific evaluations on whether the reception of the contents leads to knowledge growth are still scarce.

Research has shown the beneficial effects of public awareness and psychoeducation programs on mental disorders and stigmatization to improve knowledge and awareness of mental disorders among the general population [31, 32]. Moreover, one study evaluated the effects of an psychoeducation manual about depression in youths and reported increased knowledge and high acceptance among a sample of parents of adolescents with a history of depression [33]. There is, however, only little research on the effects of digital formats. Studies show that parents and relatives of adolescents with a history of depression report satisfaction and appreciate the helpfulness of web-based support offers [34, 35]. Previous studies have proven the efficacy of digital psychoeducation content in increasing mental health knowledge in adults and decreasing stigma related to mental disorders [36, 37]. More specifically, there is also evidence for the efficacy of internet-based psychoeducational interventions in increasing depression literacy and decreasing depression stigma in adults (e.g. [13, 38]). An RCT (randomized-controlled trial) study with parents both of healthy adolescents and adolescents with mental health problems showed that parents who took part in a web-based mental health intervention program exhibited increased knowledge about youth mental health topics and enhanced self-efficacy in dealing with these aspects compared to a waitlist control group. The intervention program contained information on the nature, symptoms and treatment of youth depression and anxiety [39]. Digital formats in outpatient treatment of mental disorders gained increasing importance during the COVID-19 pandemic. Studies on the effects of this approach during the pandemic, however, are scarce ([5], but see e.g. [40]).

Taken together, previous findings indicate that web-based formats could be both an effective and low-threshold approach to increase knowledge about mental health and depression in adults. Yet, to our knowledge, there are no studies that investigated whether imparting information about depression in childhood and adolescence via web-based formats leads to an increase in knowledge about the topic in parents. The current study aimed to close this research gap. Besides, previous studies on the effects of web-based approaches did not investigate whether individual factors, and particularly sociodemographic variables, might influence knowledge gain. This, however, is a relevant aspect as it provides insight into the question, which individuals might profit most from such approaches and it thus helps to tailor digital psychoeducational offers.

Building on the aforementioned research, the current study evaluated an innovative evidence-based information portal for parents on depression in childhood and adolescence (www.ich-bin-alles.de/eltern). The platform addresses the need for broadly available and easily accessible evidence-based information on depression and was developed with the participation of parents. The aim of the study was to investigate whether parents of adolescents with a history of depression would show increased knowledge about depression in childhood and adolescence after reception of the website and whether this knowledge gain would be stable over the course of 4 weeks. In this context, we also exploratively aimed to investigate whether sociodemographic factors would influence knowledge gain. An additional aim of the study was to assess how participants evaluate the layout and acceptance of the website. Based on previous research in adults [13, 38], we hypothesised that parents of adolescents with a history of depression would show increased knowledge about depression after presentation of the website and that this knowledge gain would be stable over the course of 4 weeks. Moreover, given the participatory approach during the development of the website, we hypothesized a positive evaluation of the portal’s layout and a high acceptance rate.

## Methods

To investigate the aforementioned study aims, i.e. (1) knowledge change after reception of contents of the website “ich bin alles”, and (2) acceptance as well as evaluation of the layout in parents of adolescents with a history of depression, a pre-post follow-up design in a convenience sample was employed. This study was preregistered on ClinicalTrials.gov (identifier: NCT05335564) and was approved by the local ethics committee.

### Participants

The sample consisted of *N* = 33 parents (*M*_age_ = 50.97, *SD*_*age*_ = 6.76, 17 females and 16 males) of adolescents (*M*_age_ = 15.61, *SD*_*age*_ = 1.62, age range 12–17 years) with a history of depression, either current or remitted or partially remitted. Information on participant’s sex was received via self-report during recruitment and in the sociodemographic questionnaire (where participants had to fill in “mother” or “father”). The history of depression was assessed via the Kinder-DIPS, a semi-structured diagnostic interview [41, 42], which was conducted with the participants’ children. The Kinder-DIPS has a good retest and interrater reliability (Cohens Kappa ≥ 0.90) [43, 44] and good validity (*p* ≤ 0.001 for the difference between target disorder and other diagnoses, indicating discriminant validity) [41]. With regard to depression diagnoses, 14 children fulfilled the criteria for a current depressive disorder (42.4%) and 19 (54.5%) were remitted or partially remitted. Participants were required to have sufficient German language skills to understand the instructions, the contents of the website and the questionnaires. A self-designed sociodemographic questionnaire was used to assess participant’s socioeconomic status index (ses-index) according to Lampert, Kroll [45]. The questionnaire contains questions on educational level, professional qualification, and income of the families. The majority of participants had a high socioeconomic status (78.8% high; 18.2% middle; 0% low; 1 missing value). For a summary of participants’ sociodemographic data, see Additional file 1: Table S1.

Participants were recruited via the Department of Child and Adolescent Psychiatry, Psychosomatics and Psychotherapy, Hospital of the Ludwig-Maximilians-University (LMU) Munich. Recruitment took place from January 2021 until April 2022. We contacted (1) parents of adolescents with a history of depression who had participated in studies on youth depression at the department in the past. In addition, we (2) recruited parents of adolescents with a history of depression who were currently undergoing treatment or had formerly been treated at the department’s clinic. During the recruitment process, we aimed to reach a balanced sample regarding participants’ sex, i.e. both mothers and fathers were contacted.

Participants were informed in detail about the procedure and aims of the study and gave written consent for their study participation. For the conduction of the Kinder-DIPS, parents of children younger than 18 years gave written consent for their children’s participation. For children aged 18, their personal written consent was sufficient. The participants received money or vouchers (worth 50€) as compensation for their participation.

## Materials

### Website

The web-based information platform “ich bin alles” (www-ich-bin-alles.de, English translation: “I am everything”) was launched in September 2021 and was developed by the Department of Child and Adolescent Psychiatry, Psychosomatics and Psychotherapy of the LMU Hospital Munich together with the Prof. Otto Beisheim Foundation and media partners. The platform contains evidence-based information on depression and mental health in childhood and adolescence. An overview on the structure and contents of the website can be found in the Additional file 1: Table S2. The information presented was developed based on extensive literature reviews as well as the S3 evidence- and consensus-based clinical guideline for the treatment of youth depression in Germany (German treatment guidelines with the highest quality level) [46]. The final website contains two sub-websites, which were specifically designed for two different target groups: (1) children and adolescents and (2) parents. The contents, language, and layout of the platform were created with the help of interviews with children and adolescents, their parents, as well as experts like child and adolescent psychiatrists and psychotherapists. The texts are written in a target-specific and appealing way and are easy to understand. For a good reading flow, the texts are structured in several sections. The information is illustrated by graphical elements. Moreover, other media formats (like videos and podcasts) are included to illustrate and complement the textual information. A professional design and media agency was involved in the conception, design and technical setup of the website.

Prior to the finalisation of the website, an evaluation website for parents, which was not publicly accessible, was created for scientific purposes. This evaluation website contained five pages of the final website with general information about depression in childhood and adolescence for parents: (1) prevalence and comorbidities; (2) professional diagnostic and treatment; (3) symptoms; (4) causes; (5) course and degree of severity. The evaluation website comprised texts, graphical elements, two videos and one podcast. In one video, for example, a child and adolescent psychiatrist answers questions on symptoms of depression in childhood and adolescence. A screenshot of the evaluation website can be found in Additional file 1: Fig. S2.

### Assessment of baseline knowledge and knowledge gain

A questionnaire designed by the research team, henceforward described as “self-designed”, was used to assess parents’ knowledge on depression in childhood and adolescence at three measurement points: their knowledge before (pre-assessment; pre) consuming the information of the evaluation website (baseline knowledge) and just afterwards at post-assessment (post) at, as well as at a follow-up testing four weeks after they had consumed the information (follow-up) (see procedure for more details). Pre- and post-assessment took place within one testing session, with the reception of the website laying in between these two time points. The knowledge questionnaire consisted of 26 items in total of which 15 used a multiple choice format (of 4 answer options, 1–4 could be correct) and the remaining 11 used a “correct or incorrect” answer format. The items corresponded to the five sections of the evaluation website (see Table 1 for an overview and item examples). Correctly answered questions were coded as 1, incorrectly answered questions were coded as 0. Thus, the final total sum score of correctly answered questions ranged from 0 to 26 across all sections (0–4 for prevalence and comorbidities; 0–2 for professional diagnostic and treatment; 0–6 for symptoms; 0–8 for causes; 0–6 for course and degree of severity).

**Table 1:** Overview of items and example items for the different sections in the knowledge questionnaire

Domain	Number of items	Example item	Answer options
Prevalence and comorbidities	4	Depression is one of the most common mental disorders in childhood and adolescence	**a) correct** b) incorrect
Professional diagnostic and treatment	2	Who can diagnose a depressive disorder?	a) anyone can assess this, if one has dealt with the topic extensively and knows the symptoms b) there are reliable tests in the internet that are helpful to find out whether one has depression or not **c) only specific professionals with appropriate qualifications can assess this** d) close friends and family can best assess this since they know the person best
Symptoms	6	What are possible symptoms of depression?	**a) sadness b) loss of interest** c) increased talkativeness **d) loss of energy**
Causes	8	If one encounters a great amount of burdens and has a genetic risk for depression, one has an increased risk to develop a depression	**a) correct** b) incorrect
Course and degree of severity	6	How are the different degrees of severity of depression called?	a) big, small b) low, strong **c) mild, moderate, severe** d) light, heavy

Correct answers are indicated in bold

### Acceptance and layout of website, and social desirability

The German short version of the Visual Aesthetics of Websites Inventory (VisAWI; [47, 48]) was used to assess participants’ evaluation of the website’s design with a focus on visual aesthetics. The VisAWI-S is a reliable (internal consistency: Cronbach’s α = 0.81) and valid questionnaire. The VisAWI-S consists of four items (e.g. “The layout appears to be designed professionally”) with a seven-point Likert scale (ranging from 1 “not agree at all” to 7 “fully agree”). The general aesthetics factor is calculated by calculating the mean value from the four item responses. As a benchmark, an overall rating of 4.5 or higher means that participants experienced the website as overall positive [49, 50]. Moreover, a self-designed evaluation questionnaire was applied to assess additional layout aspects and the acceptance of the website (for items, see Table 2). These items were assessed using a four-point rating scale (from 0 “not accurate” to 3 “entirely accurate”). Furthermore, participants were asked to give a grade for the website (ranging from 1 “very good” to 6 “insufficient”, based on the grading system used in German schools). Since participants’ tendencies towards social desirability might influence their response behaviour to questions on acceptance and layout, the Social Desirability Scale-17 (SDS-17; 51) was applied. The SDS-17 is a reliable (internal consistency: Cronbach’s α = 0.72–0.75) and valid questionnaire [51, 52].

**Table 2:** Results for evaluation questionnaire items (in %)

Items	Entirely accurate	Mainly accurate	Somewhat accurate	Not accurate
I would recommend the website to other parents.	**90.9**	9.1	0	0
I think the website is well suited for parents.	**81.8**	12.1	6.1	0
It was fun looking at the website.	**48.5**	45.5	3.0	3.0
The website is clearly structured.	**51.5**	42.4	0	3.0
I like the texts.	**60.6**	39.4	0	0
Overall, I like the pictures on the website.	**51.5**	30.3	18.2	0
I like the video.*	**54.6**	37.9	6.1	1.5
I like the podcast.	**51.5**	39.4	9.1	0

Results based on a four-point rating scale (0: not accurate, 1: somewhat accurate; 2: mainly accurate; 3: entirely accurate); %; *N* = 33; *mean values of both videos

### Procedure

Data collection was carried out from January 2021 until May 2022. Due to the COVID-19 pandemic and accompanying restrictions, the study was mostly conducted online via the secure/encrypted medical video tool RED Connect (RED Medical Systems), except for the Kinder-DIPS interviews, which were conducted in person with the affected children of the participants at the Department of Child and Adolescent Psychiatry, Psychosomatics and Psychotherapy, Hospital of LMU Munich. 31 participants participated digitally (94.0%); two participated in person (6.0%). At the beginning of the testing session, the experimenter presented the knowledge questionnaire for pre-assessment to the participants via screen-sharing. The experimenter asked the participants to provide oral answers to the questions and filled out the answers in the questionnaire. In case of participation in person at the department, participants filled out the questionnaires in paper–pencil format. After pre-assessment, the experimenter instructed the participants to open a new browser tab, type in the Website URL and a username and password. Participants were instructed to share their screen so that the experimenter could control which pages of the website participants were looking at. Thereafter, the experimenter guided the participants through the website and instructed the participants to read the textual contents of the website, and to consume the included videos and podcasts in a concentrated manner until they reached the bottom of the page. Guided by the experimenter, the participants followed a fixed procedure for each of the five website pages: “depression in childhood and adolescence” (4 min., plus a 3:27 min. video); “professional diagnostic and treatment” (3 min.); “prevalence and comorbidities (2 min.); “course and degree of severity” (3 min., plus a 7:00 min. podcast); and “causes of depression in childhood and adolescence” (6 min., plus a 4:05 min. video). In case of a participation in person, participants were seated in front of a laptop at the laboratory to look at the website. The guided instruction and the procedure for consuming the contents of the website were identical with the digital participation.

After reception of the website (post), participants were presented the knowledge questionnaire, the evaluation questionnaire, the VisAWI-S, the SDS-17 and the sociodemographic questionnaire. After a period of four weeks, the follow-up testing took place, at which the participants again were assessed with the knowledge questionnaire. 32 participants attended the follow-up digitally (97.0%), one attended in person again (3.0%). As the experimenter guided the participants through both the website contents as well as the questionnaires, the experimenter was aware of participants’ exposure history in the context of the present study.

Seven participants (21.21%) participated in the study prior to the launch of the final website. The remaining 26 participants (78.79%) participated after the launch. Therefore, at pre-assessment the latter were presented a short questionnaire assessing whether they knew the website and had already consumed the contents; moreover, at follow-up, they were asked whether they had looked at the website between post- and follow-up-assesssments. Only two particitpants indicated that they had visited the launched website briefly once between post and follow-up measurements. Sensitivity analyses showed that the pattern of results remained stable when excluding these two participants. Thus, we decided to keep these participants in the final sample. Furthermore, due to exceeded time periods between post and follow-up measurements, two participants (6 and 8 weeks between post and follow-up) had to be excluded from the analyses on knowledge changes over time, resulting in a sample of *n* = 31 adults for analysing this aspect of the study. However, they were kept in the analyses focusing on acceptance and layout (see next paragraph). For an illustration of the study procedure, see Additional file 1: Fig. S1.

### Data analysis

Statistical data analysis was carried out using IBM SPSS Statistics 26. For all analyses, the significance level was set to *p* = 0.05 (two-tailed).

To obtain knowledge scores for the three assessment points, we calculated unweighted index values for the items of the knowledge questionnaire for each participant (following [53]), separately for every domain as well as across all domains. This procedure is based on the classical test theory assumption of parallel items, which means that all items are equally good indicators for the construct that they measure. We first calculated proportional scores for each domain by dividing the sum score of the domain by the number of questions (i.e. the highest possible score) and multiplied the proportional scores with 100 to receive percentages. For the score across all domains (overall score), we summed up the proportional scores across the domains and divided this by the number of domains, and then multiplied this score with 100 to receive percentages again (see [54] for a similar approach).

To investigate changes in knowledge across domains, we conducted a repeated-measures ANOVA with time (pre/post/follow-up) as a within-subject factor. To investigate changes in knowledge for the five different domains, we conducted repeated-measures ANOVAs with time (pre/post/follow-up) and domain (prevalence and comorbidities/professional diagnostic and treatment/symptoms/causes/course and degree of severity) as within-subject factors. In case of a significant interaction effect between time and domain, we would conduct follow-up repeated-measures ANOVAs with the within-subject factor time (pre/post/follow-up) for each domain (i.e. five ANOVAs). In case of significant effects in the repeated-measures ANOVAs, we would further conduct post-hoc tests comparing the time points based on dependent *t*-tests. For the post-hoc tests, we would apply the Bonferroni-Holm correction for multiple testing and would correct all *p*-values accordingly. For all ANOVAs, we computed the effect size partial eta square (a small effect is defined as η^2^ = 0.01, a medium effect as η^2^ = 0.06 and a large effect as η^2^ = 0.16 [55]).

To exploratively investigate whether sociodemographic factors might impact baseline knowledge and knowledge change, we conducted two multiple regressions with socioeconomic status and sex as predictors and (1) baseline knowledge and (2) changes in knowledge from pre to post as criteria (i.e. the difference score between post minus pre).

To investigate participants’ evaluation of the acceptance and the layout of the website (evaluation questionnaire and VisAWI-S), we calculated descriptive statistics (*M*, *SD*). Furthermore, we conducted explorative correlation analyses (Spearman’s ρ) to investigate whether social desirability influenced participants’ answers in the evaluation questionnaire and the VisAWI-S. For these analyses, no correction for multiple comparisons was conducted, which in this case is the more conservative approach regarding the validity of our results (i.e., significant results would speak against and not in favour of the validity of our results). The analysis plan was pre-determined for the main analyses, yet not the explorative analyses.

### Power analysis

We calculated a priori power analysis to determine the necessary sample size to detect the expected effects. This calculation was based on a similar study by Kiropoulos and Griffiths [13], which investigated the effects of an internet-based information intervention in increasing depression literacy and reducing depression stigma and symptoms based on a pre-post-follow-up study by calculating ANOVAs and ANCOVAs. The authors reported large effect size for the knowledge gain from pre to post (Cohen’s *d* = − 1.78). Based on a conservative assumption of a medium effect size in the ANOVAs (*f* = 0.25), an alpha level of 0.05 and power of 0.80, the target sample size is *N* = 30. Thus, our sample of *N* = 31 was sufficiently large to detect the expected effect. The calculations for the sample size were computed with G*Power 3.1.9.2.

## Results

### Baseline knowledge

Participants’ total baseline knowledge (with *N* = 33) was *M* = 74.02% (*SD* = 11.94%) and ranged from 43 to 92% of correct answers in total. Baseline scores for the different domains were as follows: “prevalence and comorbidities” (*M* = 72.73%, *SD* = 20.12%), “professional diagnostic and treatment” (*M* = 69.70%, *SD* = 32.93%), “symptoms” (*M* = 76.77%, *SD* = 14.40%), “causes” (*M* = 73.11%, *SD* = 11.74%), and “course and degree of severity” (*M* = 77.77%, *SD* = 18.00%).

### Knowledge changes over time

**Fig. 1:**
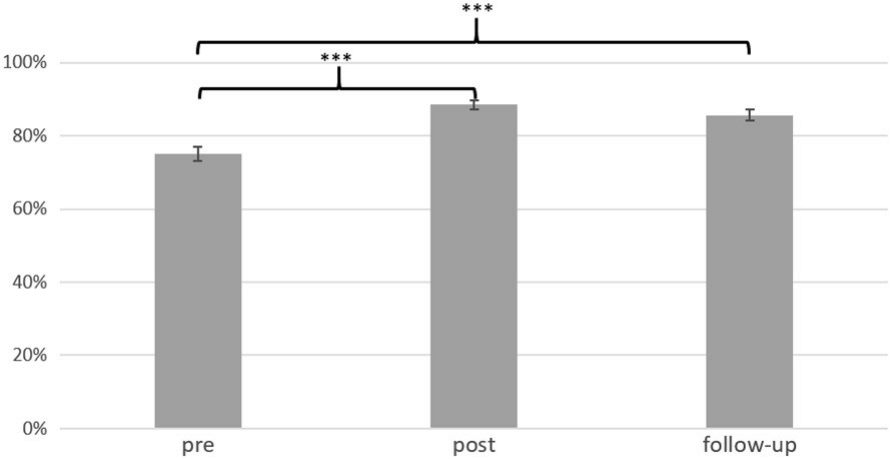
Knowledge changes over time (M, SE) in %; ***p <. 001; *n* = 31

Participants’ total knowledge (with *n* = 31) at post-assessment was *M* = 88.52% (*SD* = 7.00%) and ranged from 68 to 98% correct answers in total. Participants’ total knowledge at follow-up-assessment was *M* = 85.62% (*SD* = 8.37%) and ranged from 57 to 94% of correct answers in total. The repeated-measures ANOVA on changes in knowledge across all domains revealed a significant effect of time, *F*(2, 60) = 25.55, *p* < 0.001, partial η^2^ = 0.46. Post-hoc dependent samples *t*-tests revealed a significant increase in knowledge from pre to post (*t*(30) = − 6.55, *p* < 0.001) and from pre to follow-up (*t*(30) = − 4.95, *p* < 0.001). There was no significant change from post to follow-up (*t*(30) = 1.67, *p* = 0.106) (see Fig. 1). The repeated-measures ANOVA on changes in knowledge for the five domains revealed a significant effect of time (*F*(2, 60) = 25.55, *p* < 0.001, partial η^2^ = 0.46) and domain (*F*(2.69, 80.65) = 3.30, *p* = 0.029, partial η^2^ = 0.10). The interaction between time and domain did not reach significance (*F*(4.16, 125.07) = 1.92, *p* = 0.11, partial η^2^ = 0.06). The descriptive statistics show a similar pattern of knowledge change across all domains (see Additional file 1: Fig. S3).

### Prediction of baseline knowledge and of changes in knowledge

The regression analysis with socioeconomic status and sex as predictors and baseline knowledge as the dependent variable showed that the model with both variables did not account for a significant proportion of variance (*F*(2, 29) = 2.69, *p* = 0.085, *R*^*2*^ = 0.16). An explorative examination of the variance explained by the single predictors revealed that sex was a significant predictor of baseline knowledge (β = − 0.37; *t*(29) = − 2.08; *p* = 0.042), with mothers exhibiting a higher baseline knowledge score than fathers. By contrast, socioeconomic status did not account for a significant proportion of variance.

The second regression analysis with socioeconomic status, sex and changes in knowledge from pre to post as the dependent variable revealed that the variables did not account for a significant proportion of variance (*F*(2, 29) = 0.23, *p* = 0.794, *R*^*2*^ = 0.02). Explorative examination showed that neither of the two variables had a significant effect on changes in participants’ knowledge (see Additional file 1: Table S3 for an overview of the regression analyses).

### Evaluation of the layout and acceptance of the website

The average value of the VisAwi-S general aesthetic factor was *M* = 5.83 (*SD* = 0.88) and thus exceeded 4.5 as the benchmark for a positive evaluation of the website’s layout. The overall grade given to the website was *M* = 1.76 (*SD* = 0.55), and thus fell in between very good and good. The results for participants’ answers to the evaluation of the website are presented in Table 2.

Correlation analyses revealed a significant negative correlation (Spearman’s ρ = -0.48, *p* = 0.005) between participants’ social desirability tendency and the VisAWI-S general aesthetic factor. Thus, against what might be expected, higher levels of social desirability were associated with lower scores in the VisAWI-S. Yet, there were no significant correlations between participants’ social desirability tendencies and the items of the evaluation questionnaire (all *p*s ≥ 0.116).

## Discussion

The main goal of the study was to investigate whether parents of adolescents with a history of depression show increased depression literacy after the reception of a website about youth depression and whether this increase remains stable at follow-up. Another main aim of the study was to assess the reception of the layout and acceptance of the website in the study sample. We found an increase in overall knowledge from pre to post that remained stable from post to follow-up. Given the non-significant interaction between time and domain, this pattern was evident across domains. The reception of the layout was positive, as were the acceptance ratings for the website. Imparting and increasing knowledge through low-threshold psychoeducational approaches like websites are particularly relevant in times of crisis and increased prevalence rates of depressive symptoms and disorders.

Our findings of a stable increase in knowledge with large effect sizes provide promising support that our digital approach can educate parents of adolescents with a history of depression. Our findings are in line with previous studies on the effects of (online) information formats in increasing knowledge on mental health and depression, and decreasing stigma in adults (e.g. [13, 22, 31, 33, 36–38]).

Despite high baseline knowledge rates, participants’ knowledge gain was significant with a mean of 13.5% from pre to post and 10.6% from pre to follow-up. As a digital information portal is a low-threshold offer that can reach many people, even numerically small changes might be clinically relevant when they result in more treatment seeking and subsequently more beneficial outcomes in adolescents with depression. Yet, it was beyond the scope of the present investigation to assess a transfer of the acquired knowledge into everyday life or the potential benefits on clinical outcomes, which should be addressed in future studies.

With respect to the results of the regression analyses, it first needs to be discussed that sociodemographic variables did not influence the extent of knowledge gain. This suggests that both mothers and fathers from adolescents with a history of depression with different socioeconomic backgrounds seem to profit from our online information portal. Yet, it has to be noted that the majority of our participants had a high socioeconomic status and nones had a low socioeconomic status. Further research with a more diverse sample in terms of sociodemographic status is therefore needed in order to replicate our results and draw more comprehensive conclusions.

Although the regression analysis on the influence of sociodemographic variables on baseline knowledge failed statistical significance, it is worth noting that—on an explorative level—mothers exhibited a higher baseline knowledge than fathers. This goes in line with findings of previous research showing that women exhibit higher mental health literacy (e.g. [56, 57]) and are more informed about mental illnesses than men [58]. Another explanation for the higher baseline knowledge of mothers might be that, typically, they are still more involved in their children’s upbringing. Hence, mothers might have gathered more knowledge about their child’s depression, e.g. in the context of its treatment and other contacts to the health care system than fathers (e.g. [59]). Taken together, our explorative finding emphasizes the importance of targeting not only mothers but in particular fathers to close knowledge gaps.

The positive evaluation of the layout of the website and our finding of high acceptance based on the evaluation questionnaire fits into previous findings that show a high acceptance of digital information platforms on mental disorders [29, 30]. High acceptance rates and e.g. confirmatory responses regarding the tendency to recommend the website to others are essential for online formats to reach as many people as possible and to ensure the dissemination of the contents in the target groups. In this context, it’s worth briefly commenting on the outreach of the final website, which attracts around 1000 daily visitors 2-years post-launch (analysed via the tool Matomo). Together with the promising results regarding the evaluation, this emphasizes the potential of the participative approach taken during the development of our website, with the perspective and feedback of the target groups being strongly considered. Our findings on the relationship between the evaluation results and social desirability clearly speak against the notion that the positive reception of the website was biased by tendencies to respond socially desirable. Indeed, we even found a negative correlation between higher social desirability tendencies and the reception of the website’s layout as assessed with the VisAWI-S.

Web-based ehealth services, like our digital information portal, bear many benefits for their defined target groups. They provide an easily and broadly accessible possibility to get important information on mental health contents, including information on professional services. Since research has shown that, in parents of children and adolescents with a history of depression, higher knowledge regarding mental disorders, symptom severity and treatment is associated with more treatment-seeking, improved treatment decision and better treatment quality concerning their children, low-threshold information offers might contribute to more beneficial outcomes [17–19]. However, not all adolescents receive the parental support they would need. Thus, adolescents themselves are in need of target-specific, comprehensive information on mental health content. To meet this need, next to the parents’ sub-website of “ich bin alles”, the website contains a sub-website for adolescents, which was evaluated in two independent studies (pre-registered at ClinicalTrials.gov: NCT05300204; NCT05300217).

Our results of increased knowledge and stable knowledge change after the reception of contents of a web-based information portal on youth depression provide an important basis for similar approaches and might stimulate further research on web-based portals, which is still scarce. Furthermore, in light of the promising findings regarding the high acceptance of the portal and the positive evaluation of its layout, the approach chosen could inform the design of other web-based portals on mental health issues. Next to research related implications, the results of our study might also bear implications for policy decisions concerning the relevance of ehealth services in mental health education and campaigns.

As the study was conducted during the COVID-19 pandemic, it might be the case that the restrictions and burdens associated with the crisis have influenced the results on the acceptance and evaluation of the website, in that information offers on mental health contents were perceived as particularly relevant. Yet, also after the end of pandemic-related restrictions, the website attracts a high number of visitors, thus proving support for its acceptance in the naturalistic setting.

## Limitations and conclusions

Some limitations of the study need to be considered. The majority of the parents had a high socioeconomic status, which might be attributed to the fact that the sample was drawn from a convenience sample. Therefore, the generalizability of the results should be supported in future studies including a population with a lower socioeconomic status. This is of particular importance, as these families are of greater risk for developing mental health problems (e.g. [3]) and should thus be particularly considered in the context of low-threshold offers. Moreover, the study population was drawn from a convenience sample consisting of parents of adolescents with a history of depression who were currently or had been in treatment at the clinic for child and adolescent psychiatry of the Hospital of the LMU Munich. Therefore, generalizability of the results to other parent groups, including e.g. parents of untreated offspring, should be supported in future studies. In this context, it should be said that we have carried out another study with parents of healthy adolescents (ClinicalTrials.gov: NCT05326178), the results of which will be reported elsewhere.

Furthermore, the study employed a single-armed pre-post-follow-up design, which does not exclude the possibility that factors that are not related to the reception of website contents per se contributed to knowledge change (including e.g. the readministration of the questionnaires or the possibility that participants got information elsewhere). Yet, between pre- and post-assessment, the study was conducted in a highly controlled laboratory setting, where participants were under continuous supervision, thus excluding the possibility of parents getting information elsewhere between these measurement points. This having said, future studies with a randomized-controlled study design are clearly needed to draw more rigorous conclusions on the efficacy of the website.

Despite the a priori power analysis, which indicated that the sample size was sufficiently large to detect the expected effects, a sample of *N* = 33 represents a rather small number. Future studies should thus recruit larger samples to draw even more robust conclusions. Related to the restricted sample size, the present study could only exploratively investigate the possible influence of sociodemographic factors. In order to draw more rigorous conclusions on the influence of sociodemographic factors on knowledge change, larger samples are clearly warranted.

Despite these limitations, this is the first study on knowledge effects and acceptance of a digital information platform on youth depression in a sample of parents of adolescents with a history of depression. Our study provides important information for the design of digital platforms addressing similar topics like “ich bin alles”. Digital offers on mental health are a low-threshold approach to inform parents and increase their knowledge and are thus especially important in times of limited resources or increased need such as the COVID-19 crisis. Moreover, approaches like ours might also decrease stigma. Given the negative consequences of youth depression when untreated (e.g. [3, 15]) as well as the important role of parents in supporting their children (e.g. [17, 18]), it is essential to promote information platforms that are broadly available and easily accessible. Future studies should systemically explore whether such approaches also improve early detection and effective treatment of depression in affected youths.

### Supplementary Information

**Additional file 1: Table S1. **Sociodemographic Data. **Table S2.** Contents of the final website. **Figure S1. **Study procedure. **Figure S2.** Screenshot of the evaluation website on the contents of prevalences & comorbidities. **Figure S3.** Differences in knowledge changes over time (in %). **Table S3.** Results of the regression analyses; N = 33.

## Data Availability

Our data includes sensitive patient information, such as information on comorbidities. Since participants could possibly be identified by making our raw data publicly available, ethical principles of protecting patient confidentiality would be breached. Thus, raw data cannot be made publicly available. We can make additional materials and aggregated data available upon request.

## Abbreviations

ADHD: Attention-deficit/hyperactivity disorder
ANOVA: Analysis of variance
Follow-up: Follow-up assessment
Kinder-DIPS: Diagnostic Interview for Mental Disorders for Children and Adolescents
LMU: Ludwig-Maximilians-University
M: Mean
Pre: Pre-assessment
Post: Post-assessment
RCT: Randomized-controlled trial
SD: Standard deviation
SDS-17: The Social Desirability Scale-17
SE: Standard error
SES: Socioeconomic status
