# Supplementary material for: Evaluation of a web-based information platform on youth depression and mental health in parents of adolescents with a history of depression
Source: Child Adolesc Psychiatry Ment Health. 2024 Jan 13;18:7. doi: 10.1186/s13034-023-00703-x (PMC10787406; doi:10.1186/s13034-023-00703-x)
Supplement: Supplementary file 1 — Additional file 1: Table S1. Sociodemographic Data. Table S2. Contents of the final website. Figure S1. Study procedure. Figure S2. Screenshot of the evaluation website on the contents of prevalences & comorbidities. Figure S3. Differences in knowledge changes over time (in %). Table S3. Results of the regression analyses; N = 33. [file 13034_2023_703_MOESM1_ESM.docx]

**Additional file 1**

**Sociodemographic data**

| *Table S1*  *Sociodemographic Data* | |
| --- | --- |
| Age (year)  *M (SD)* | 50.97 (6.76) |
| Sex  *Female, n (%)*  *Male, n (%)* | 17 (76.2)  16 (23.8) |
| MDD status of child  *Current, n (%)*  *Remitted, n (%)* | 14 (42.4)  19 (54.5) |
| SES  *middle, n (%)*  *high, n (%)* | 6 (18.2)  26 (78.8) |

**Structure and Contents of the final website**

| *Table S2*  *Contents of the final website* | |
| --- | --- |
| Topic | Subtopics |
| Detecting depression | - **Symptoms of depression in children and adolescents** - **Diagnostic process** - **Experts for diagnostic and treatment of youth depression** |
| Understanding depression | - **Course of depression and degrees of severity** - **Prevalence rates and comorbidities** - **Causes of youth depression** - Stress factors   - Refugee experiences   - Alcohol, cigarettes and illegal drugs   - Family   - School and social field   - Traumatic/crucial life events |
| Maintaining mental health | - Supporting child’s mental health - Promoting a positive parent-child relationship - Helping to deal with problems - Helping to deal with stress - Helping to deal with social media - Dealing with (cyber)mobbing |
| Treating depression | - Search for therapists and clinics - In-patient stay - S3-treatment guidelines (Germany) - Treatment options (setting) - Treatment recommendations, depending on degree of severity - Recommended psychotherapies - Recommended medication |
| Supporting affected persons | - Organising everyday life - Shaping parent-child relationship - Interacting with a depressive child |
| Help | - Overview of professional help offers for parents |
| Note*. Contents used for the evaluation website are indicated in bold.* | |

**Procedure**


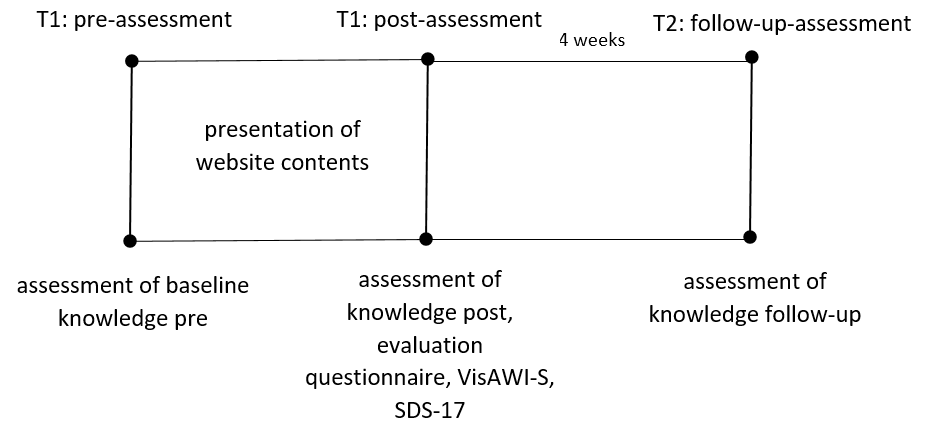


Figure S1. *Study procedure.*

**Screenshot of the evaluation website**

**
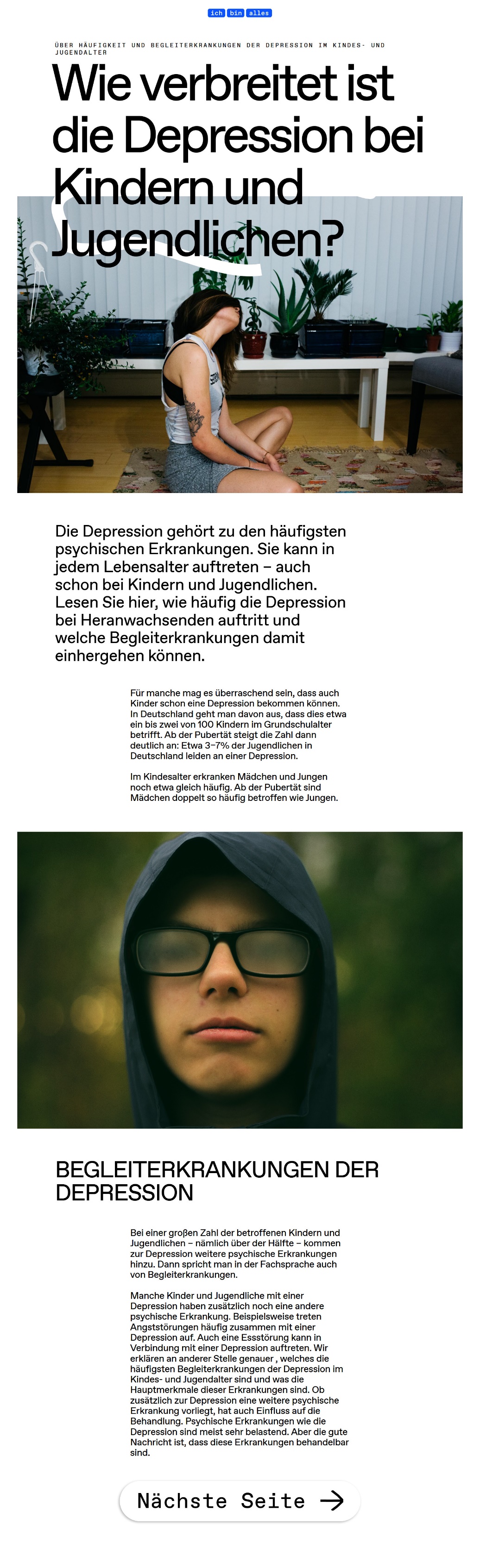
**

**
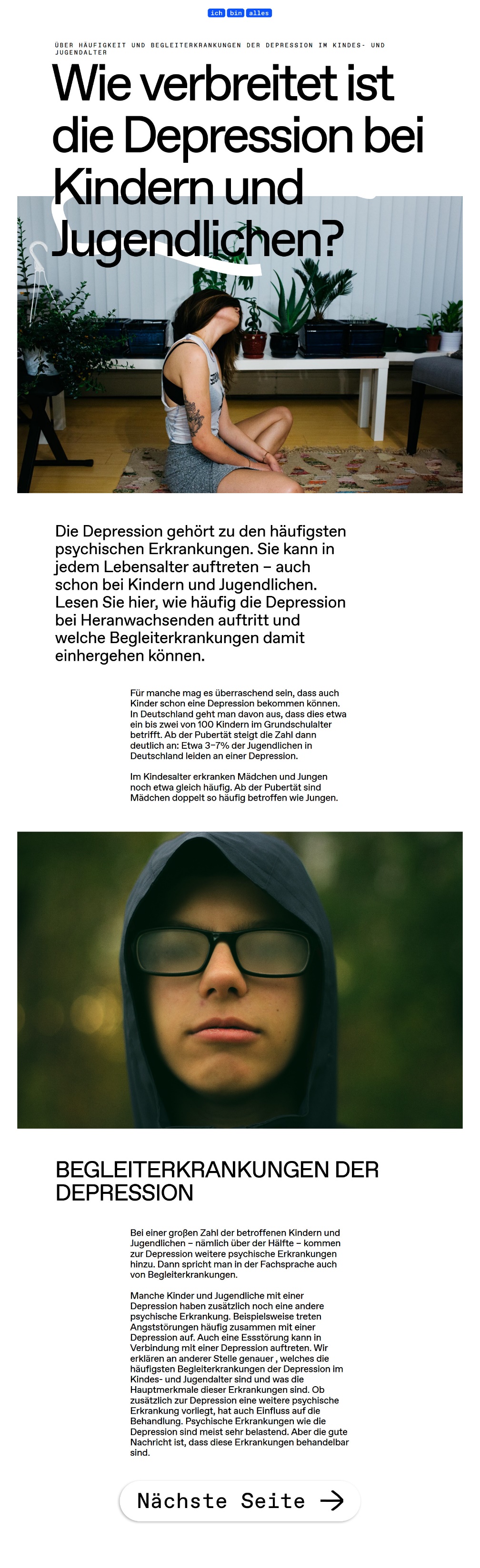
**

Figure S2. *Screenshot of the evaluation website on the contents of prevalences & comorbidities.*

**Domain-specific knowledge changes over time**

**
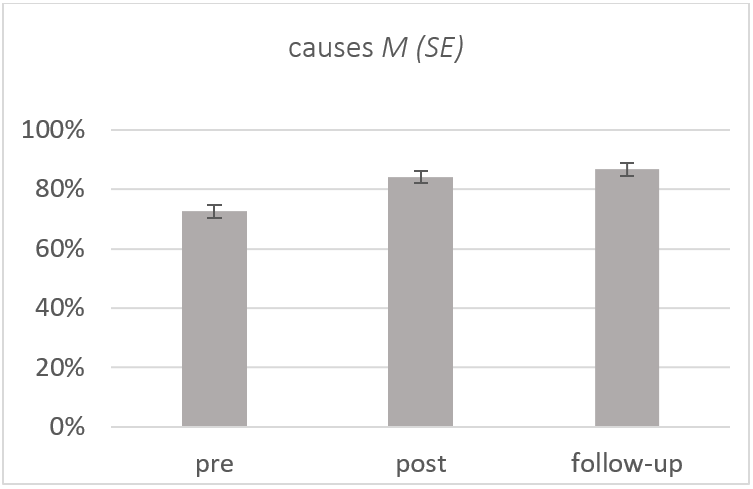

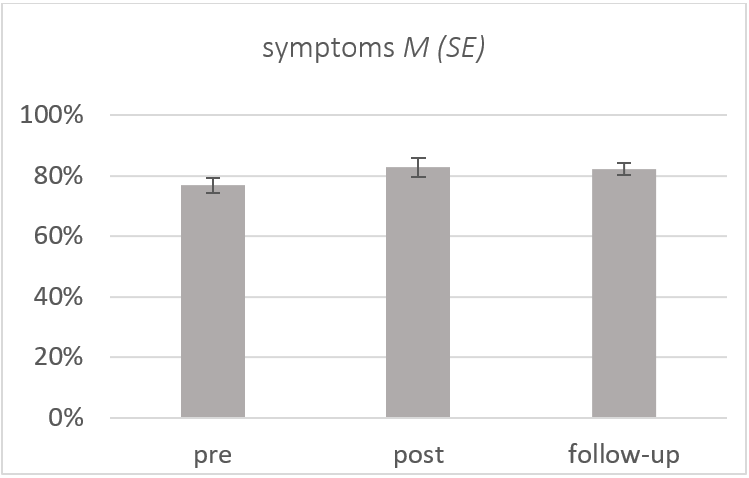

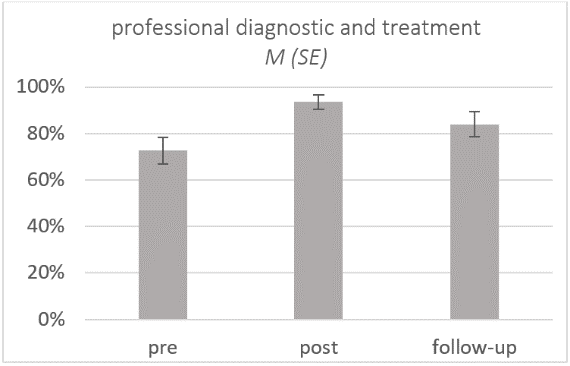

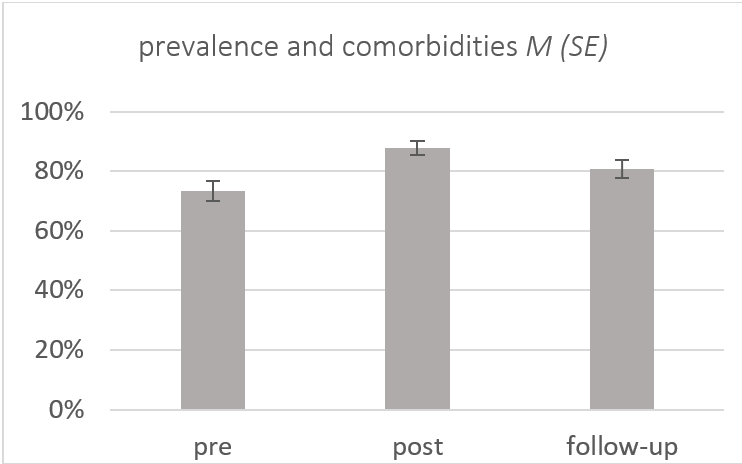
**(*F*(4.16, 125.07) = 1.92, *p* = .11, partial η² = .06):

**
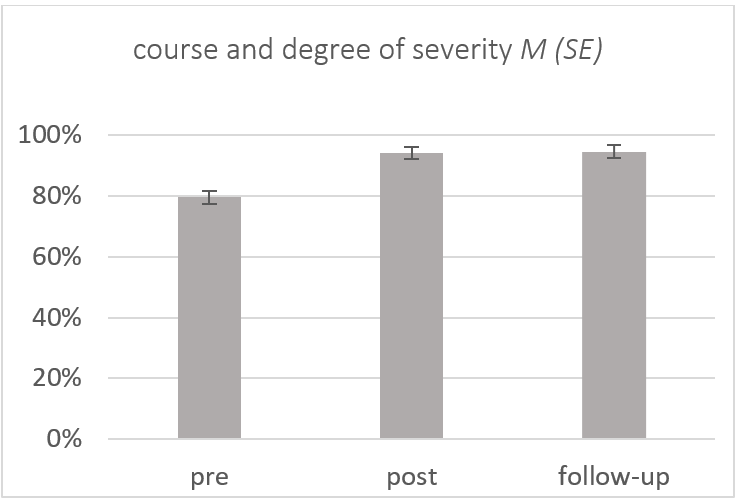
**

Figure S3. *Differences in knowledge changes over time (in %);*

*N = 31. M = mean; SE = standard error.*

| **Regression analyses** | | | | | | | |
| --- | --- | --- | --- | --- | --- | --- | --- |
| *Table S3*  *Results of the regression analyses; N = 33* | | | | | | | |
|  | | *B* | *SE for B* | *95 % CI for B* | *β* | *t* | *p* |
| ***Baseline knowledge*** |  | |  |  |  |  |  |
| Sex | -0.09 | | 0.04 | [-0.17, -0.01] | -.37 | -2.08 | < .05 |
| Socioeconomic status | 0.09 | | 0.05 | [-0.25, -0.20] | .28 | 1.59 | .124 |
| ***Knowledge change pre/post*** | | | | | | | |
| Sex | 0.03 | | 0.04 | [-0.06, 0.12] | .12 | 0.62 | .540 |
| Socioeconomic status | -0.03 | | 0.06 | [-0.14, 0.09] | -.09 | -0.45 | .656 |
